# Adjuvant Radiotherapy Improved Survival in Stage I to II Low-Grade Endometrial Stromal Sarcoma: A Retrospective Study of 152 Cases

**DOI:** 10.3389/fonc.2020.608152

**Published:** 2021-01-22

**Authors:** Wenhui Wang, Shuai Sun, Zheng Miao, Xiaorong Hou, Fuquan Zhang, Ke Hu

**Affiliations:** Department of Radiation Oncology, Peking Union Medical College Hospital, Chinese Academy of Medical Sciences and Peking Union Medical College, Beijing, China

**Keywords:** low-grade, endometrial stromal sarcoma, adjuvant radiotherapy, stage I to II, prognosis

## Abstract

**Objective:**

Low-grade endometrial stromal sarcoma (LG-ESS) is a rare gynecological tumor. Whether adjuvant radiotherapy benefits survival in patients with resected early-stage ESS remains controversial. This study was designed to explore the role of adjuvant radiotherapy in stage I to II LG-ESS.

**Methods:**

We retrospectively reviewed patients with stage I to II LG-ESS in our center from Jan. 1998 to Feb. 2018. All patients underwent a total hysterectomy and postoperative radiotherapy was administrated based on clinical and pathological characteristics.

**Results:**

A total of 152 patients with stage I to II resected LG-ESS were included. Forty patients received adjuvant radiotherapy (RT group) while 112 patients did not receive adjuvant radiotherapy (no RT group). The baseline characteristics of the two groups were comparable, except that the proportion of stage II patients in the RT group was higher than that in the no RT group (32.5% vs. 11.6%, in RT vs. no RT groups, respectively; p = 0.003). For both patient groups, median overall survival was not reached. The median disease-free survival (DFS) was 144 months. Radiotherapy was associated with significantly improved DFS (92 months vs. not reached in RT vs. no RT groups, respectively; p = 0.008) and pelvic failure-free survival (PFFS) (92 months vs. not reached in RT vs. no RT groups, respectively; p=0.004). Subgroup analysis revealed that RT benefited survival most among patients with stage IB to IIB disease. Adjuvant radiotherapy significantly reduced the pelvic recurrence rate (10.0%, 4/40 vs. 28.6%, 32/112, p = 0.018). No radiotherapy-induced grade 4 to 5 toxicity was observed.

**Conclusion:**

For patients with stage I to II LG-ESS, adjuvant radiotherapy showed significant improvement in DFS and PFFS with tolerable adverse effects, especially in patients with stage IB to IIB disease.

## Introduction

Uterine sarcoma (US) is a malignant mesenchymal tumor that accounts for approximately 1% of female genital tract malignancies and 3% to 7% of all uterine tumors (1, 2). US includes low-grade endometrial stromal sarcoma (LG-ESS), high-grade endometrial stromal sarcoma, undifferentiated uterine sarcoma, and uterine leiomyosarcoma. LG-ESS typically has an indolent clinical course, with 5- and 10-year survival rates of 90% or higher (3, 4). Hysterectomy remains the current standard of care. However, after surgery, the pelvic recurrence rate in patients with ESS is as high as 10%–30% (3, 5, 6) indicating that current approaches might not be sufficient to completely eradicate pelvic disease.

Radiotherapy on postoperative LG-ESS patients consists of external beam radiotherapy to the pelvis to eliminate microscopic disease in the pelvic area and intracavitary brachytherapy to the vaginal cuff. The role of adjuvant radiotherapy (RT) is controversial. Due to the rarity of LG-ESS, no data about the efficacy of adjuvant RT are available from prospective randomized controlled clinical trials (7). Existing retrospective literature about postoperative radiotherapy consists of single-institution reviews that are limited by the heterogeneity of selection criteria and treatments. This study was designed to evaluate the efficacy of RT on the long-term outcomes of patients with early-stage low-grade endometrial stromal sarcoma in a high-volume center.

## Materials and Methods

### Patients

Patients with stage I to II LG-ESS who underwent hysterectomy with or without bilateral salpingo-oophorectomy between Jan. 1998 and Feb. 2018 were retrospectively analyzed. Patients were restaged based on from the 2009 International Federation of Gynecology and Obstetrics (FIGO) for uterine sarcomas criteria. We retrospectively reviewed medical records for age, performance status, site of primary tumor, tumor size, pathological stage, surgical procedure, and treatment approaches. Patients with the following clinical scenarios were excluded: uterine sarcoma other than low-grade ESS, stage III to IV, palliative resection or myomectomy, RT dose less than 45 Gy, and preoperative radiotherapy or chemotherapy. The study was approved by the Institutional Review Board of Peking Union Medical College Hospital (No. S-K1193).

### Treatment Approaches

All patients underwent total hysterectomy in stage I and resection of extrauterine disease in stage II. Bilateral salpingo-oophorectomy (BSO) was strongly recommended for LG-ESS patients especially when ER/PR was positive. However, oophorectomy was individualized for reproductive-age patients. Lymphadenectomy was performed for patients with preoperative image showing enlarged lymph nodes, or suspicious enlarged lymph nodes during operation, or for patients with extrauterine disease. Based on the preference of the surgeon and the pathological characteristics and physical status of the patient, radiotherapy was performed using a 6 MV X-ray linear accelerator and intensity modulated radiotherapy (IMRT), conventional radiotherapy (CRT), or three-dimensional conformal radiotherapy (3D-CRT) modalities. The radiation field used for conventional radiotherapy covered the pelvic area and part of the vagina using the four-field “box” technique. For IMRT and 3D-CRT methods, the clinical target volume covered the upper part of the vagina and regional lymphatic drainage regions, including the common iliac, internal iliac, external iliac, obturator, and presacral areas. A margin of 0.6–1 cm was added to the planning target volume to account for organ motion. The radiation dose was prescribed to the isocenter or 95% planning target volume as 45.0–50.4 Gy in 20–28 fractions. A dose of 10–20 Gy in 2–4 fractions was administered to the upper part of the vaginal stump with high dose rate brachytherapy. Radiation toxicities were evaluated using Common Terminology Criteria for Adverse Events Version 4.0 (CTCAE 4.0).

Adjuvant hormonal therapy was recommended especially to patients at premenopausal age or without BSO for half a year to five years. However patient initiation and compliance with hormonal therapy was inconsistent due to concerns about side effects. Hormone therapy included aromatase inhibitors, megestrol acetate, or medroxyprogesterone acetate, and gonadotropin-releasing hormone analogs. Intravenous adjuvant chemotherapy consisted of PE (cisplatin/epirubicin), PEI (cisplatin/epirubicin/ifosfamide), PI (cisplatin/ifosfamide), and PVB (cisplatin/vincristine/bleomycin).

### Data Analysis

Survival durations were defined as the time from surgery to: the date of death due to any cause (overall survival, OS); the date of treatment failure or death due to any cause (disease-free survival, DFS); or the date of pelvic failure or death due to any cause (pelvic failure-free survival, PFFS).

Data was analyzed using SPSS statistical software (version 25.0; SPSS Inc., Chicago, IL). The chi-square test was used for categorical variables. For continuous variables, normality tests were performed using the Kolmogorov-Smirnov method. The Student’s *t* test was used to assess normally distributed variables. The Kaplan-Meier method was used to calculate survival data, and differences between groups were determined using the log-rank test. Propensity-matched analysis (PSM) was used to eliminate group differences. Patients in the RT group were matched one-to-one to those in the no RT group. A Cox regression model was used to estimate the treatment effect within each patient subgroup. A p-value of < 0.05 was considered statistically significant.

## Results

### Patients and Tumor Characteristics

A total of 152 patients with stage I to II (FIGO 2009) LG-ESS were analyzed. Patient characteristics are summarized in **Table 1**. All patients had Karnofsky performance scores over 80. The median age of patients was 44 years (range, 21–68 years). Most patients (134/152, 88.2%) were premenopausal at disease onset. All patients underwent total hysterectomy. Bilateral salpingo-oophorectomy was performed in 77.6% (118/152) of patients, while ovary preservation was achieved in the remaining 34 patients. Eighteen of the 34 underwent total hysterectomy because of preoperative diagnosis of uterine fibroids and second surgery was refused by patients despite the diagnosis of LG-ESS. Fourteen cases were young premenopausal patients. Preoperative imaging examination (such as MRI) demonstrated stage I disease, and they strongly demanded ovarian function preservation. Two patients preserved ovaries because of unknown reasons. Lymphadenectomy was performed in 17.8% (27/152) of patients. The median tumor diameter was 6.0 cm (range, 1.4–17.0 cm). Most patients were in FIGO stage I (126/152, 82.9%, including stage IA, 39/152 and stage IB, 87/152). Patients in FIGO stage II accounted for 17.1% (26/152) of all patients.

**Table 1:** Baseline clinical characteristics of patients treated with and without radiotherapy.

Clinical Characteristic	Patients (N=152)						
	Total (n=152)		RT (n=40)		No RT (n=112)		*p*-value
	No.	%	No.	%	No.	%	
**Age, years**							0.737
Mean	43.1		43.5		42.9		
Range	21–68		21–66		21–68		
**Stage (FIGO)**							0.003
I	126	82.9	27	67.5	99	88.4	
II	26	17.1	13	32.5	13	11.6	
**Menstruation status**							0.315
Premenopausal	134	88.2	33	17.5	101	9.8	
Postmenopausal	18	11.8	7	82.5	11	90.2	
**Pathologic tumor size, cm**							0.427
mean	6.5		6.8		6.4		
Range	1.4–17		3.0–15.0		1.4–17.0		
**Ovary preservation**							0.081
Yes	34	22.4	5	12.5	29	25.9	
No	118	77.6	35	87.5	83	74.1	
**Hormonal therapy**							0.155
Yes	69	45.4	22	55.0	47	42.0	
No	83	54.6	18	45.0	65	58.0	
**Chemotherapy**							0.451
Yes	14	9.2	2	5.0	12	10.7	
No	138	90.8	38	95.0	100	89.3	

RT, radiotherapy; FIGO, International Federation of Gynecology and Obstetrics.

After surgery, 40 patients (26.3%) received adjuvant radiotherapy (RT group), and 112 patients (73.7%) did not (no RT group). Hormone therapy was administered in 45.4% (69/152) of patients. Adjuvant chemotherapy was performed in 9.2% (14/152) of patients. Baseline characteristics were comparable between the two groups (p > 0.05), except that the proportion of patients with stage II disease (32.5% vs. 11.6%, for RT vs. no RT groups; p = 0.003) was significantly higher in the RT group. In the RT group, 27 patients received IMRT, two patients received 3D-CRT, and 11 patients received CRT. Thirty-two patients received both external irradiation and brachytherapy, while eight patients were only administered external irradiation. The use of brachytherapy was preferred but predominantly at the physicians discretion accounting for distance to intestinal tract or patients who are not sexually active. The median external radiation dose prescribed was 50.4 Gy (range, 45.0–50.4 Gy), and the median brachytherapy dose was 10 Gy (range, 0–20 Gy).

### Predictors of Survival

For all patients, the median follow-up time was 52 months (range, 2-239 months). Two patients in the no RT group died at 17 and 43 months after surgery because of disease recurrence. Median OS was not reached, and the 1-, 3-, 5-, and 10-year OS rates were 100%, 99.2%, 98.2%, and 98.2%, respectively. The median DFS was 144 months, and the 1-, 3-, 5-, and 10-year DFS rates were 93.9%, 82.0%, 73.4%, and 58.3%, respectively.

Univariate survival analysis showed that radiotherapy, postmenopausal status, and bilateral salpingo-oophorectomy significantly prolonged DFS. The Cox multivariate regression model revealed that radiotherapy and bilateral salpingo-oophorectomy were significant factors affecting DFS.

### Effects of Radiotherapy on Survival

**Figure 1:**
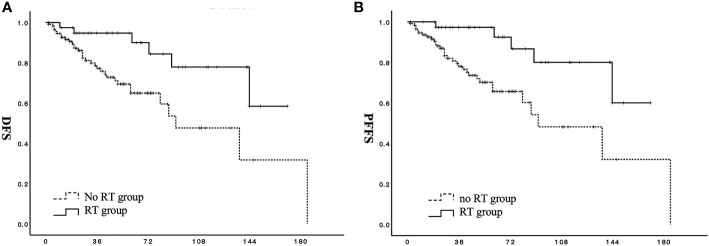
Effect of adjuvant radiotherapy on survival for patients with stage I to II low-grade endometrial stromal sarcoma. **(A)** DFS, disease-free survival; **(B)** PFFS, pelvic failure-free survival; 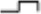 RT group; 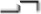 no RT group.

For all patients in the RT group, adjuvant radiotherapy significantly improved DFS (p = 0.008) and PFFS (p = 0.004) (**Figures 1A, B**). For patients in the RT group, the 3-, 5-, and 10-year DFS rates were 94.7%, 94.7%, and 71.9%, respectively. The 3-, 5-, and 10-year DFS rates for patients in the no RT group were 77.2%, 64.9%, and 47.6%, respectively. The OS values of patients in the two groups did not significantly differ (p = 0.378).

**Figure 2:**
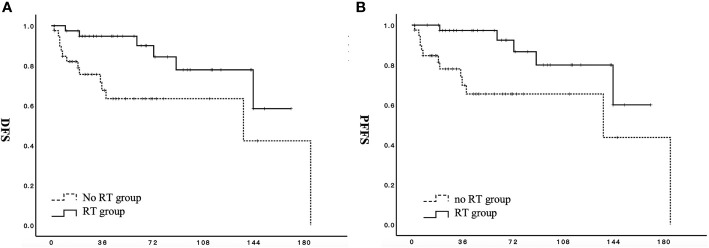
Effect of adjuvant radiotherapy on survival for patients with stage I to II low-grade endometrial stromal sarcoma after propensity-matched analysis. **(A)** DFS, disease-free survival; **(B)** PFFS, pelvic failure-free survival; 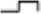 RT group; 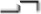 no RT group.

The proportion of patients with stage II disease was significantly higher in the RT group than in the no RT group. Therefore, a stage-based propensity matching analysis was feasible for 80 patients (13 stage II patients and 27 stage I patients from each group). Other factors, including age, menstrual status, ovarian preservation rate, and endocrine therapy, were comparable. The results of this analysis reveal that DFS (p = 0.020) and PFFS (p = 0.015) are significantly better in the RT group than in the no RT group (**Figures 2A, B**).

**Figure 3:**
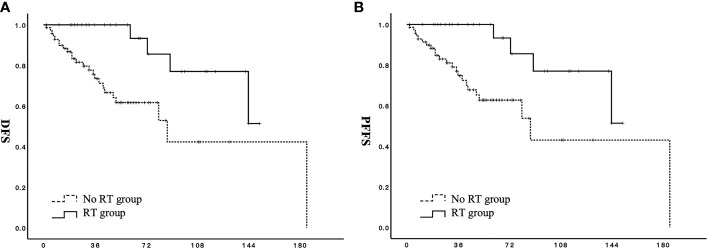
Effect of adjuvant radiotherapy on survival for patients with stage IB to IIB low-grade endometrial stromal sarcoma. **(A)** DFS, disease-free survival; **(B)** PFFS, pelvic failure-free survival; 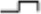 RT group; 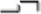 no RT group.

Stage-based subgroup analysis showed improved DFS in the RT group among patients with stage IB to IIB (HR 0.224; 95% CI: 0.085–0.593) (**Figure 3A**). RT was also associated with prolonged PFFS in patients with stage IB to IIB (HR 0.180; 95% CI: 0.062–0.525) (**Figure 3B**).

The fact that all patients completed the prescribed dose of adjuvant radiotherapy was taken into account when assessing the toxicity of irradiation. The most common acute adverse effects reported were enterotoxicity (Grade 1–2, 22.5%; Grade 3, 7.5%) followed by hematological toxicity (Grade 1–2, 7.5%). In the RT group, there were no Grade 4 or above toxicities during the treatment or follow-up periods.

### Failure Pattern

Thirty-nine relapses (25.7%) were identified. The main failure pattern was pelvic failure (36/152, 23.7%), followed by distant (9/152) and abdominal failure (7/152). The median time to recurrence was 28 months (range, 2 to 185 months). Of all relapses, only three patients only had distant relapses, 25 were limited to the pelvic area, and the other 11 were in the pelvic area with abdominal or distant sites.

The pelvic failure rate was significantly lower in the RT group (4/40, 10.0%) than in the no RT group (32/112, 28.6%) (p=0.018). Examination of the type of adjuvant radiotherapy received by the four RT group cases with pelvic recurrence revealed that IMRT tended to reduce the pelvic relapse rate (1/27, 3.7%) compared with non-IMRT techniques (3/13, 23.1%) (P = 0.056).

## Discussion

### Main Findings

For patients with FIGO (2009) stage I to II LG-ESS, adjuvant radiotherapy significantly improves DFS and PFFS and reduces the pelvic failure rate with tolerable toxicity. Subgroup analysis revealed that in patients with stage IB to IIB disease, adjuvant radiotherapy was most beneficial for DFS and PFFS. To the best of our knowledge, this report includes the largest sample size of any studies focusing on the efficacy of adjuvant radiotherapy in patients with early-stage LG-ESS and exploring the role of radiotherapy based on risk factors.

### Interpretation of Results

Endometrial stromal sarcoma accounts for 21% of all uterine sarcomas (8) and can be subdivided into distinct low- and high-grade entities based on histopathology, clinical behavior, and patient outcomes (9). For early-stage LG-ESS, the National Comprehensive Cancer Network consensus guidelines recommend surgical resection. Adjuvant radiotherapy is classified as an appropriate intervention for women with stage II or late disease without enough evidence (2).

The role of radiation therapy in localized LG-ESS is controversial. LG-ESS is rare, so most LG-ESS data arises from epidemiologic studies involving the outcomes of all uterine sarcomas (10). The only published prospective randomized phase III study performed by the European Organization for Research and Treatment of Cancer included stage I and II uterine sarcoma (103 leiomyosarcoma, 91 carcinosarcomas, and 28 ESS). However, no survival benefit was observed in the RT group compared to the control group of leiomyosarcoma and ESS patients (6). Two retrospective studies included a relatively large number of LG-ESS cases. Zhang et al. (5) analyzed 104 LG-ESS patients. Among them, 31 received radiotherapy. The results showed that radiotherapy was related to worse PFS. However, details of radiotherapy were not illustrated and the relatively small number of patients with radiotherapy might weaken the conclusion. Zhou et al. (3) conducted a retrospective analysis of 114 patients with LG-ESS, 36 of which received RT. In this study it was predicted that adjuvant radiotherapy did not significantly increase DFS, but only 78% of the included patients were diagnosed with stage I to II disease.

Some retrospective studies suggested that adjuvant RT was associated with better local-regional control and improved survival. Sampath et al. (11) analyzed 351 cases of ESS in the National Cancer Database and found that, for patients receiving adjuvant radiotherapy, the 5-year local-regional RFS was significantly higher than that in patients not receiving adjuvant radiotherapy (P < 0.05). However, these reports did not distinguish between high-grade and low-grade ESS. Valduvieco et al. (12) conducted a retrospective study including 13 cases of ESS. They concluded that the local control rate of postoperative radiotherapy was significantly higher in patients that received RT than in those that had not. In some retrospective studies, radiotherapy was recognized as a protective factor for progression free survival (13, 14). However, other studies concluded that adjuvant RT appeared to be of limited clinical value in LG-ESS (15, 16).

Due to small participant numbers, most retrospective reports have grouped uterine sarcoma subtypes into a single category. For studies that only include ESS, results are often not reported by grade. Moreover, most retrospective studies have shown considerable prognostic imbalances between irradiated and nonirradiated patients. This might be because radiotherapy is often administered to patients with poor prognoses. Therefore, these data are insufficient for interpretation. Large national database inquiries often lack specifics about the therapy administered, in-depth demographic information, and tumor details and other retrospective studies include heterogeneous disease types and small numbers. Our results provide an inclusive homogenous analysis with detailed clinicopathologic factors. Only stage I to II low-grade ESS cases were included in this study. Characteristics were comparable between the two groups except that the frequency of FIGO stage II was significantly higher in the RT group than in the no RT group (p = 0.003). A PSM analysis was performed to eliminate group differences, and showed that RT significantly improved DFS and PFFS. Late stage is identified as a risk factor in some retrospective studies (5, 13, 17). Our stage-based subgroup analysis showed that RT was most beneficial for patients with stage IB to IIB.

As this tumor has a long natural history and pelvic radiation could carry significant late toxicity, other treatment options should be highlighted. Efficacy of hormonal therapy among completely resected early stage LG-ESS has not been confirmed beyond doubt. As an adjuvant ablative form of hormonal therapy, the role of oophorectomy remains unclear while some retrospective researches supported its benefit in pelvic control. Zhang et al. (5) demonstrated hormonal therapy was a protective factor with respect to PFS among LG-ESS patients. In Leath’s (18) research, results showed patients treated with endocrine treatments tended to have a higher median OS. However, in a retrospective series among 114 LG-ESS patients, ovarian preservation and endocrine therapy had no significant effect on DFS. According to ESMO-EURACAN guidelines (19), given retrospective evidence suggesting its role in decreasing relapses, adjuvant hormonal therapy might be an option among early stage LG-ESS patients. According to NCCN guidelines, estrogen blockade was recommended in the 2B category for stage I patients. In our research, statistics demonstrated hormonal therapy tended to improve DFS and bilateral salpingo-oophorectomy was a significant factor affecting DFS. As an indolent tumor, efficacy of adjuvant radiotherapy should be weighed against its side effects, such as secondary malignancy risk, especially in young woman. Administration of adjuvant hormonal therapy should be paid attention to especially in a young population where pelvic radiation could carry significant late toxicity, such as secondary malignancy risk.

The literature on the ESS failure pattern is limited. Zhou et al. (3) demonstrated that failure of LG-ESS was mainly due to distant metastasis (64.3%, 9/14) and only 5/14 recurrences were pelvic. However, 22% of the patients included their study were in an advanced stage. Leath et al. (18) reported that the most common site of initial failure for low-grade ESS was the pelvis (11/72, 15%). Previous studies have shown that the local-regional recurrence rate, after curative resection, ranges from 10–35% (3, 5, 6). In this study, pelvic failure was the dominant ESS failure pattern (23.7%) followed by distant and abdominal failure. Patients in the RT group had a lower pelvic failure rate than did those in the no RT group (p = 0.018).

IMRT has been a widely applied radiation technique over the last ten years. Compared with CRT and 3D-CRT, IMRT has higher conformity and uniformity, which reduces adverse reactions for organs at risk. In this study, patients receiving IMRT had a lower pelvic recurrence rate than did those receiving CRT or 3D-CRT. However, efficacy of IMRT should be observed after a long-term follow-up. RT appears to provide local tumor control and the prognosis of patients with LG-ESS is usually good, so the benefit of adjuvant RT should be weighed against its side effects (20). In this study, no grade III or above radiation-induced intestinal or urinary tract toxicity was identified during treatment or follow-up.

### Strengths and Limitations

There are some limitations to this study. First, as a retrospective, single-center study, there might be selection bias in inclusion. Second, adverse effects might be underestimated because of the retrospective nature of the study and the fact that most patients were outpatients. Third, this study population was evaluated over a 20 year time period. The standard management of tumors over this length of time could have changed leading to heterogeneity of radiation modality and surgical management. Besides, long time span made it hard to assess the tumor control issue when recurrence happened late. Lastly, the number of patients in the RT group was much lower than that in the no RT group (40 patients vs. 112 patients for RT and no RT groups, respectively), which might weaken our findings and conclusions. Despite these limitations, this is the largest population-based study exploring adjuvant radiotherapy in resected early-stage LG-ESS patients and could be a valuable reference that provides guidance for RT selection in specific subgroups.

## Conclusion

For patients with FIGO (2009) stage I to II LG-ESS, adjuvant RT significantly reduces the pelvic failure rate and improves DFS and PFFS with tolerable toxicity. This is especially true in patients with stage IB to IIB disease, and makes adjuvant RT a preferred adjuvant treatment option. Further validation of these results is warranted in the future.

## Data Availability Statement

The raw data supporting the conclusions of this article will be made available by the authors, without undue reservation.

## Ethics Statement

The studies involving human participants were reviewed and approved by Institutional Review Board of Peking Union Medical College Hospital (No. S-K1193). Written informed consent for participation was not required for this study in accordance with the national legislation and the institutional requirements.

## Author Contributions

WW participated in the conceptualization, data curation, and writing the original draft. SS contributed to validation of the results. ZM developed the methodology and performed formal analysis. XH reviewed and edited the manuscript. FZ performed project administration. KH supervised the research. All authors contributed to the article and approved the submitted version.

## Funding

This study was supported by the Non-profit Central Research Institute Fund of Chinese Academy of Medical Sciences (grant number 2019XK320046) and the Ministry of Science and Technology of the People’s Republic of China (grant number 2016YFC0105207). The funding bodies had no role in study design, subject enrollment, or data analysis.

## Conflict of Interest

The authors declare that this research was conducted in the absence of any commercial or financial relationships that could be construed as a potential conflict of interest.

## Abbreviations

LG-ESS, low-grade endometrial stromal sarcoma; LG, Low-grade; HG, high-grade; US, uterine sarcomas; OS; overall survival; DFS, disease free survival; PFFS, pelvic failure-free survival; 3D-CRT, three-dimensional conformal radiotherapy; mOS, median overall survival time; mDFS, median disease-free survival time; RT, radiotherapy; IMRT, intensity modulated radiation therapy; CTV, clinical target volume; PTV, planning target volume; FIGO, International Federation of Gynecology and Obstetrics; CTCAE 4.0, Common Terminology Criteria for Adverse Events Version 4.0; WPRT, whole pelvic radiotherapy; SEER, Surveillance, Epidemiology, and End Results Database; EORTC, European Organization for Research and Treatment of Cancer.
